# EMDL-ac4C: identifying N4-acetylcytidine based on ensemble two-branch residual connection DenseNet and attention

**DOI:** 10.3389/fgene.2023.1232038

**Published:** 2023-07-13

**Authors:** Jianhua Jia, Zhangying Wei, Xiaojing Cao

**Affiliations:** School of Information Engineering, Jingdezhen Ceramic University, Jingdezhen, China

**Keywords:** ac4C site identification, ensemble deep learning, DenseNet, attention mechanism, residual structure

## Abstract

**Introduction:** N4-acetylcytidine (ac4C) is a critical acetylation modification that has an essential function in protein translation and is associated with a number of human diseases.

**Methods:** The process of identifying ac4C sites by biological experiments is too cumbersome and costly. And the performance of several existing computational models needs to be improved. Therefore, we propose a new deep learning tool EMDL-ac4C to predict ac4C sites, which uses a simple one-hot encoding for a unbalanced dataset using a downsampled ensemble deep learning network to extract important features to identify ac4C sites. The base learner of this ensemble model consists of a modified DenseNet and Squeeze-and-Excitation Networks. In addition, we innovatively add a convolutional residual structure in parallel with the dense block to achieve the effect of two-layer feature extraction.

**Results:** The average accuracy (Acc), mathews correlation coefficient (MCC), and area under the curve Area under curve of EMDL-ac4C on ten independent testing sets are 80.84%, 61.77%, and 87.94%, respectively.

**Discussion:** Multiple experimental comparisons indicate that EMDL-ac4C outperforms existing predictors and it greatly improved the predictive performance of the ac4C sites. At the same time, EMDL-ac4C could provide a valuable reference for the next part of the study. The source code and experimental data are available at: https://github.com/13133989982/EMDLac4C.

## 1 Introduction

RNAs from both eukaryotic and prokaryotic cells may include a broad range of nucleoside modifications (Tardu et al., 2019), and it is statistically known that there are more than 170 types (Boccaletto et al., 2018). Song et al. (2023) have developed the RMDisease database and identified a large number of disease-associated variants to elucidate the important regulatory role of RNA modifications. Among them, N4-acetylcytidine (ac4C) is a highly conserved RNA modification, and at the same time, he is the sole acetylation modification of eukaryotic RNA that has been identified (Zhao et al., 2019a; Jin et al., 2020). Ac4C plays an important role in biology, and it has different functions on different RNAs. On tRNA, ac4C helps to improve the accuracy of protein translation and maintain the heat resistance of the organism (Kumbhar et al., 2013); the role of ac4C on rRNA likewise includes maintaining high fidelity of protein translation (Sharma et al., 2015), while it is also a marker of thermophilic organisms, which is significant; on mRNA, ac4C is required to safeguard the stability of mRNA while increasing the efficiency of protein translation (Arango et al., 2018; Dominissini and Rechavi, 2018). Meanwhile, Chen et al. (2022) demonstrated that the only known ac4C writer, N-acetyltransferase 10 (NAT10), has an important effect in male reproduction. In addition, ac4C has regulatory effects on viruses (Tsai et al., 2020; Hao et al., 2022) and has been associated with several human diseases, including: osteoporosis (Yang et al., 2021), pancreatic cancer (Feng et al., 2022), *etc.*

The recognition of ac4C sites has gradually become a popular topic in biology and computer research. In the context of biological experiments, multiple testing methods exist for ac4C studies. Previously, partially enzymatic hydrolysis and two-dimensional paper chromatography were commonly used to identify ac4C modifications in RNA (Thomas et al., 2019). In the past few years, researchers had found that the combination of LC-MS and HPLC-MS analyses is more efficient in isolating partially modified nucleic acids, including ac4C (Ito et al., 2014; Sharma et al., 2015; Sharma et al., 2017). In addition, RPHPLC (Mezzar et al., 2014) is widely used by the biological community as it requires only a small number of samples and does not rely on expensive mass spectrometry assays or the use of radioactive substrates. There are also specific ac4C sequencing approaches, including ac4C-seq (Gamage et al., 2021) and RedaC:T-seq (Sturgill et al., 2022), both of which sequence ac4C through a series of experiments under certain conditions. Nevertheless, these experimental methods always have several problems, such as time-consuming and high expensive.

Several machine learning-based predictive models (Zhou et al., 2016; Wei et al., 2019; Basith et al., 2019; Lv et al., 2020; Hasan et al., 2021) for the predictive identification of RNA post-translational modification sites have been developed by researchers in the past several years. Among them, there are two predictors used to identify ac4C sites, firstly, Zhao et al. (2019a) developed PACES based on position-specific dinucleotide sequences as well as K-nucleotide frequency coding, which was trained using two random forest classifiers. Secondly, Alam et al. (2020b) proposed XG-ac4C predictor based on this, which used multiple encoding methods, including one-hot, nucleotide chemistry and density, Kmer, *etc.*, and used extreme gradient boosting (XGboost) to train the dataset to identify ac4C loci. Nonetheless, neither of these two predictors' ability in making predictions is sufficient.

With the widely use of deep learning, researchers have introduced different deep learning models to the field of RNA post-transcriptional modification site prediction (Yu and Dai, 2019; Liu et al., 2020; Hasan et al., 2021; Rehman et al., 2021; Hasan et al., 2022; Tsukiyama et al., 2022; Yang et al., 2023), and a lot of experimental results show that deep learning models perform better than machine learning models for datasets with a large number of samples. Muhammad et al. (Iqbal et al., 2022) used a deep learning network-based CNN model using an encoding approach similar to the XG-ac4C predictor DL-ac4C predictor was proposed to identify ac4C sites, and compared with machine learning methods, the results showed that DL-ac4C has better prediction performance. Recently, a new deep learning model DeepAc4C (Wang et al., 2021a) had also been developed to increase the efficiency of ac4C locus recognition in mRNA. It also used CNN to extract information and classify the feature maps, and encoded a combination of physicochemical features and nucleotide semantic information. Yet, the classification performance of these two predictors still needs to be improved. As a result, a more analytically precise model to anticipate ac4C sites is urgently required.

In order to more accurately predict ac4C sites in mRNAs, an effective prediction model EMDL-ac4C was developed in this paper, and the contributions of this paper were various: 1) EMDL-ac4C used only the simplest encoding method one-hot to represent nucleotides. This encoding showed the distribution probability of each nucleotide, making it easier to calculate the distance between nucleotides. 2) It proposed a powerful deep learning that used DenseNet in combination with convolutional residuals to form two-branch residual connection DenseNet, and the effectiveness of feature extraction was increased by this way. 3) For cases like this paper, where unbalanced datasets were processed into multiple balanced datasets, downsampling integration was used to achieve significantly superior generalization performance than a single learner. 4) The attention mechanism was carried out throughout the network structure to give greater attention to the important information at each stage. 5) We compared the performance of various encoding techniques, different numbers of dense blocks, multiple model architectures, and several predictors, respectively, to confirm the efficacy of the EMDL-ac4C model.

Therefore, the final predictor is called “EMDL-ac4C,” in which “EM” stands for “ensemble,” “DL” represents “deep learning,” and “ac4C” means “N4-acetylcytidine."

## 2 Materials and methods

### 2.1 Benchmark dataset

The base dataset for this study was extracted from 2,134 genes offered by Zhao et al. (2019a) from a highly throughput dataset previously presented. In the training set, there were a total of 1,160 positive and 10,855 negative samples, and in the test set, the counts of positive and negative samples were: 469, 4,343, respectively. Alam et al. (2020b) also built the XG-ac4C predictor from this dataset. Wang et al. (2021b) performed a de-sampling redundancy with a threshold of 0.4 using CD-HIT (Weizhong et al., 2006) software in order to remove redundant sequences from these datasets, resulting in 1,615 positive and 7,590 negative samples. These samples were separated into a set for training and one for testing, with 1,148 positive samples and 5,439 negative samples in the former. On the other hand, there were 467 positive samples and 2,151 negative samples in the independent test set. Finally, they created ten balanced datasets using the unbalanced training and test sets, respectively, to make it easier to train and test the model. The number of positive samples in each sub-training set and test set remains the same, and negative samples are randomly selected from the corresponding negative dataset, and the number is consistent with the positive sample size. The ten samples are denoted as D1, D2, , D10. Table 1 shows an example of the distribution of data set D1: (the distribution of D2, D3, , D10 is the same).

**TABLE 1:** Distribution of data set D1.

Dataset	Positive	Negative
Training	1,148	1,148
Testing	467	467

As in Eq. 1, nucleotide sequences containing potential N4-acetylcytidine sites can normally be read as:

$$f_{\delta }\left(K\right)=R_{-\delta }R_{-\left(\delta -1\right)}\cdots R_{-2}R_{-1}KR_{+1}R_{+2}\cdots R_{+\left(\delta -1\right)}R_{+\delta }$$ (1)

where the center K denotes “N4-acetylcytidine,” $R_{-\delta }$ means the δth upstream nucleotide from the center K, while $R_{+\delta }$ stands for the *δ*th downstream nucleotide from the center K. In this study, *δ* is 207, that is, the length of a nucleotide sequence is (2*δ* +1).

### 2.2 Feature coding methods

#### 2.2.1 One-hot coding

The RNA sequence used in this study consists of four nucleotides and a “-,” where the “-“ represents a missing value or an undetected nucleotide in the RNA sequence. One-hot encoding, also known as binary encoding, converts each nucleotide into a numeric vector of 0 and 1, encoding “A” as [1, 0, 0, 0, 0], “C” as [0, 1, 0, 0, 0], “G” as [0, 0, 1, 0, 0], “T” as [0, 0, 0, 1, 0], and “-” is assigned as [0, 0, 0, 0, 0, 1]. One-hot encoding not only simply converts sequence information into digital information for computer processing, but more importantly, it makes the calculation of distances between nucleotides more reasonable. For example, if nucleotides are represented in sequential encoding: 1:A, 2:C, 3:G, 4:T, then the distance between A (adenine ribonucleotide) and C (cytosine ribonucleotide) is smaller than the distance between A (adenine ribonucleotide) and G (guanine ribonucleotide), which is not reasonable. At the same time, one-hot coding in fact means the probability distribution of nucleotides, that is, one nucleotide has probability 1 and the others are all 0.

### 2.3 Classification model

#### 2.3.1 Base learning model

**FIGURE 1:**
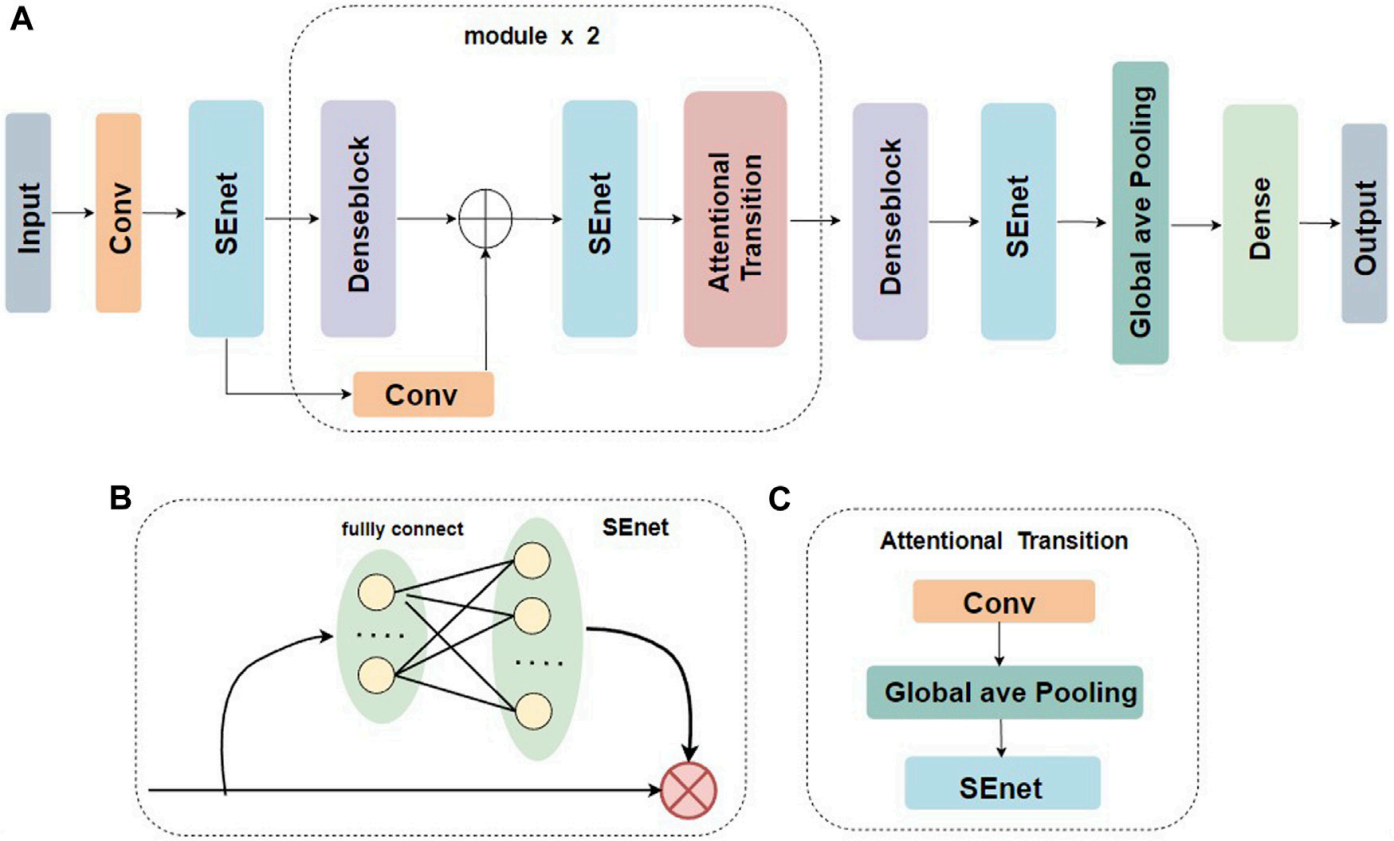
Base classifier for EMDL-ac4C. **(A)** Schematic graph of the two-branch residual connection DenseNet model. **(B)** Schematic diagram of the SEnet module. **(C)** Schematic diagram of the Attentional Transition module.

Figure 1 illustrates the model structure of the base learner two-branch residual connection DenseNet for constructing EMDL-ac4C. A): The basic structure of two-branch residual connection DenseNet consists of SENet, denseblock, attentional-transition, residual convolution layer and fully connected layer. B): SENet is composed of two fully connected layers with different number of neurons to fulfill the aim of first dimensionality reduction and then dimensionality increase of the feature map. C): The attentional-transition is composed of 1 × 1 convolution, global average pooling layer and SENet, where the role of convolution and global average pooling is to condense the output feature map of two-branch denseblock and convolutional residual module, decrease the size and dimension of the feature map, and simultaneously can successfully decrease the quantity of denseblock parameters and stop the network to excessive fitting. The purpose of SENet is to extract important features. The final fully connected layer is used as the classification prediction of the model.

Below are explanations of each step in greater detail.

##### 2.3.1.1 DenseNet

Huang et al. (2017) proposed DenseNet in 2017 for the target recognition task to alleviate the gradient disappearance issue that often occurs in deep networks, while feature reuse also enhanced feature propagation with fewer parameters in a network of equal layer depth. As shown in Eq. 2, ResNet only adds features to the input of the latter layer and connects them in a summation manner. DenseNet is an improvement of ResNet in that it combines the features of each layer by concatenation. As shown in Eq. 3, DenseNet connects all the previous layers as the input of the next layer, obtaining better performance than ResNet with fewer parameters and computational cost.

$$x_{l}=H_{l}\left(x_{l-1}\right)+x_{l-1}$$ (2)

$$x_{l}=H_{l}\left(\left[x_{0},x_{1},\ldots ,x_{l-1}\right]\right)$$ (3)

The $H_{l}\left(•\right)$ in Eqs 2, 3 represents the non-linear transformation function, which is a combined operation that may include a series of BN (Batch Normalization), ReLU(Rectified Linear Unit), POOL (Pooling) and Conv (Convolution) operations. The non-linear transformation function in this paper adopted the structure of BN+ReLU+1*1 Conv +3*3 Conv, which were created through a preactivation strategy to facilitate network training as well as enhanced the efficiency of generalization, and 1 × 1 Conv served to reduce the number of features, thus reduced computational workload and improved computational efficiency. In addition, 3 × 3 Conv offered a larger receptive field.

**FIGURE 2:**
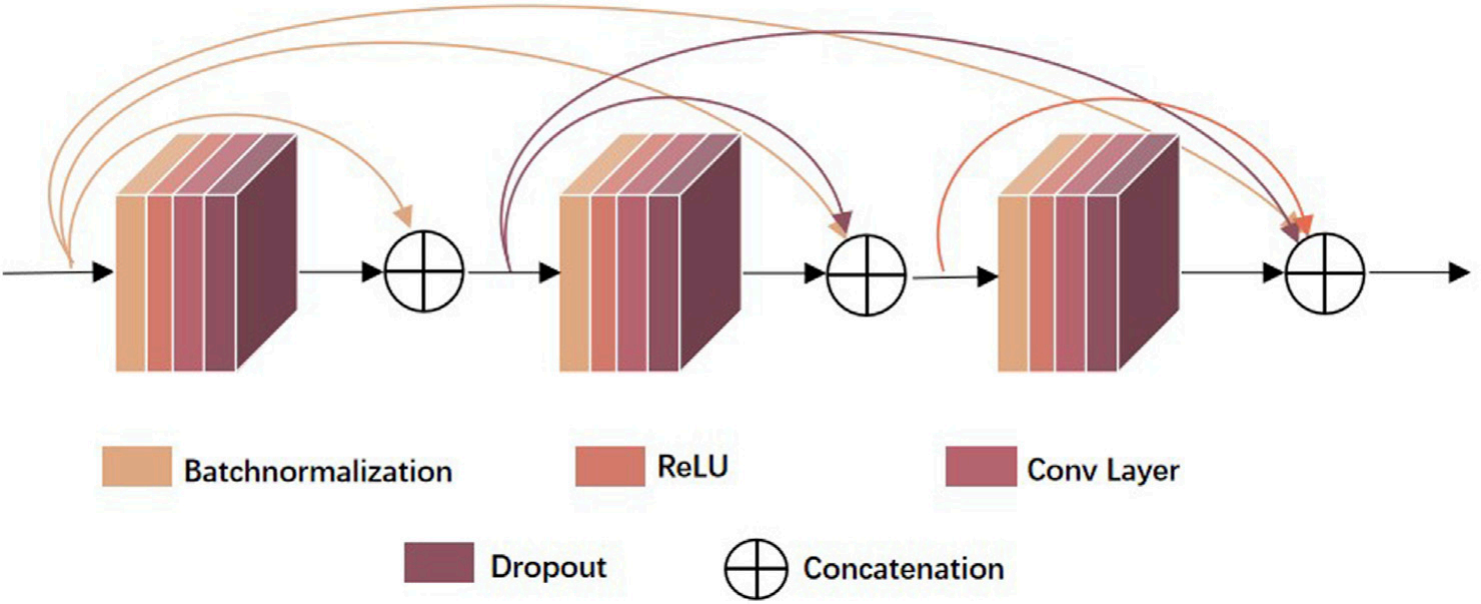
The structure of denseblock.

As shown in Figure 2, the denseblock is a module containing many layers, each layer has the same feature map size, and the layers are closely connected to each other, while the Transition module connects two neighboring denseblocks and reduces the feature map size by Pooling. In this paper, we used a new approach Attentional Transition instead of transition, whose construct is depicted in Figure 1C.

##### 2.3.1.2 Residual connection

In order to effectively spread shallow information to deep layers, the output of residual connection is represented by a linear superposition of the inputs and their nonlinear transformations. Deep learning propagates the gradient (derivative) of the loss function step by step from the back to the front in the back propagation process, and the gradient is less than 1 at each level. Due to the cumulative multiplicative effect, the gradient may be too small and cause the network to stop training optimization. Therefore, the addition of residual connections to the network can address the issue of network degradation and enhance network functionality.

**FIGURE 3:**
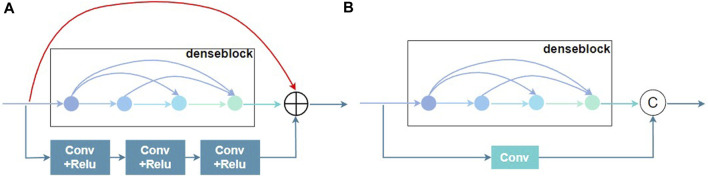
Residual connection schematic of the denseblock module. **(A)** RXDNFuse’s multi-branch Residual denseblock. **(B)** Two-branch Residual denseblock.

Long et al. (2021) proposed the aggregated residual dense network (RXDNFuse) for the mix problem of IR and visible images, which combined the residual and convolutional residuals into parallel dense blocks to extract multi-level features, as depicted in Figure 3A. The results also demonstrated that RXDNFuse can effectively retain the important thermal radiation information in the feature map, from which this work was inspired to propose Two-branch residual connection dense network, which consisted of a denseblock and a convolutional residual connection to form two channels. As illustrated in Figure 3B, compared with RXDNFuse, two-branch residual connection DenseNet has three differences, firstly, it reduces one layer of residual connection, secondly, it changes convolutional residual connection part from 3 layers of Conv+ReLU to one layer of Conv to extract features, and for the last, it changes the residual connection method from summation to concatenation. In this way, we can improve the diversity of feature extraction and achieve the effect of two-branch feature extraction, while reducing the complexity of the model.

##### 2.3.1.3 SENet

Introducing attention mechanism in the predictor can make model more efficient in learning the interrelationships between feature information (Vaswani et al., 2017), while focusing on useful information. (Wang et al., 2020; Wang et al., 2021; Jia et al., 2022; Jia et al., 2023). The use of the attention mechanism in both image processing and natural language learning has demonstrated its value in enhancing the model’s capacity for recognition, and our study again confirms this point that the attention mechanism can help suppress useless information, pay attention to critical information, and improve the model performance.

Researchers have developed various attention mechanisms, commonly used including self-attention mechanisms, spatial attention mechanism, channel attention mechanism, and so on. Squeeze-and-Excitation Networks (SENet) (Hu et al., 2018) is one of the channel attention mechanisms. We investigated the use of attention mechanisms in our model in two ways, one was to choose the attention mechanism that best matches the model and the data characteristics, and try to use the four attention mechanisms individually or in combination. The second was where to place the attention mechanism in the model. There are various options for where to insert attention into the model, including introducing it in densecells (Bastings and Filippova, 2020), adding it in denseblocks (Wei et al., 2019; Zhou et al., 2019; Wang et al., 2021), inserting it between denseblocks and transitions (Jia et al., 2022; Jia et al., 2023), bring in the attention mechanism in the transition layer (Song et al., 2021), or attaching it before the data enters DenseNet or at the end of the model prediction.

The combined use of SENet and DenseNet has been repeatedly shown to boost network detection and site prediction performance (Yan et al., 2019; Wang et al., 2021; Shi et al., 2021; Jia et al., 2023). We had also found after numerous ablation experiments that SENet alone works best, while it was advisable to place SENet before DenseNet, between denseblock and Attentional Transition, and before the final global average pooling, as seen in Figure 1A. Adding the attentional layer before the initial feature map enters the DenseNet helps the model not miss important information in the original feature map, while the attentional layer behind denseblock aims to repress redundant features and strengthen propagation of important features.

The construction of SENet is to first perform a global pooling operation on the feature map with input h*w*c, which is a spatial compression process that makes the feature map 1*1*c in size. Next there are two fully connected layers. The first full connection has c/16 neurons, which is a dimensionality reduction procedure, and the second fully connected is ascending to c neurons. The significance of dimensionality reduction and then dimensionality increase is to discover the correlation between channels. The final step is to multiply the original h*w*c feature map with the 1*1*c feature map after dimensionality down and dimensionality up to obtain a feature map with the importance levels of different channels.

It is worth noting that the SENet used in this paper removes the global pooling operation at the beginning, the reason is that the global pooling operation will lose some location information.

##### 2.3.1.4 Attentional transition

For the transition layer, it mostly connects two neighboring denseblocks and decreases the size of the feature map. Yang et al. (Yang et al., 2018) proposed CliqueNet to alleviate the training challenge of deep networks, which introduced a channel-based attentional mechanism in the transition layer. Following their approach, we also introduce the attentional mechanism in transition layers to ensure high-quality propagation of features between dense blocks. As Figure 1C shows, after convolution, the feature map is then brought into SENet, which means that the feature map is first global average pooled, followed by two fully connected layers (FC) to complete the descending and ascending operations.

#### 2.3.2 Ensemble learning

**FIGURE 4:**
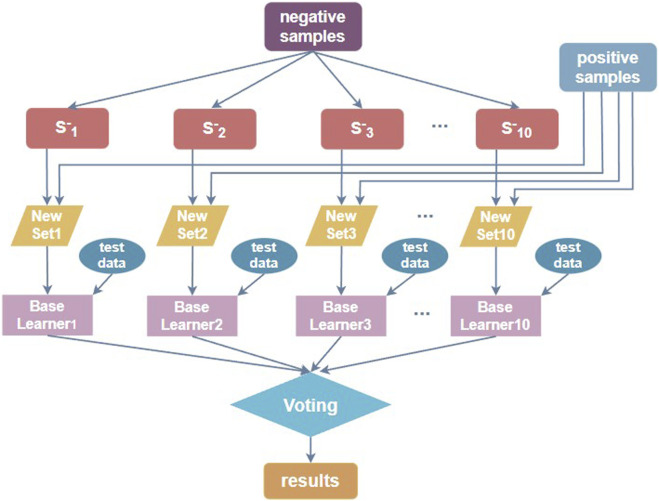
Downsampling ensemble classifier construction process.

When the number of positive samples in the benchmark dataset is significantly lower than the number of negative sample data, the dataset is unbalanced. For such a non-equilibrium dataset, using a simple model is not friendly to identify positive samples, while for us, the information of N4-acetylcytidine loci is the most critical and the most necessary to be identified. The ensemble learning method can be used to downsample the non-balanced data, meaning that for the majority of negative samples, downsampling is performed each time, and the same number of subsets as positive samples are extracted, and the two constitute a balanced dataset for training. Multiple sub-classifiers are thus constructed, and then the training models are validated by cross-validation and the model training effect is verified by independent test sets, respectively. Such use of the ensemble classifier dramatically improves the accuracy of loci prediction (Jia et al., 2016). The process of constructing the downsampling ensemble classifier is shown in Figure 4.

Wang et al. (Wang et al., 2021) constructed the dataset in a similar manner to Figure 4, with ten random downsampling of negative samples to build ten balanced datasets. Therefore, we used 10 balanced training sets to build 10 sub-classifiers, trained the model by cross-validation, and then validated the model training effect using ten independent test sets one by one. The prediction results obtained from multiple sub-classifiers were soft-voted to obtain the final ensemble results. The base sub-classifiers are implemented with the two-branch residual connection DenseNet shown in Figure 1A.

#### 2.3.3 Performance evaluation

Five metrics are typically used to evaluate models in such studies: Accuracy (Acc), Sensitivity (Sn), Specificity (Sp), Area under curve (AUC) and Matthew’s correlation coefficient (MCC), which are computed as follows in Eq. 4:

$$\begin{array}{c} Sn=\frac{TP}{TP+FN}\\ Sp=\frac{TN}{TN+FP}\\ Acc=\frac{TP+TN}{TP+TN+FP+FN}\\ MCC=\frac{TP\times TN-FP\times FN}{\sqrt{\left(TP+FN\right)\times \left(TN+FN\right)\times \left(TP+FP\right)\times \left(TN+FP\right)}} \end{array}$$ (4)

TP is a correctly identified positive ac4C site, FN is a misidentified positive ac4C site, and TN and FP are correctly and incorrectly predicted negative ac4C sites, respectively. Researchers often use ROC curves to indicate the performance of classifiers, and the area under the ROC curve is measured as the AUC value, and a bigger value indicates greater performance.

In this study, we used these five prevalent evaluation metrics to assess the performance of EMDL-ac4C.

## 3 Results and discussion

### 3.1 Comparison of models with different denseblocks

**FIGURE 5:**
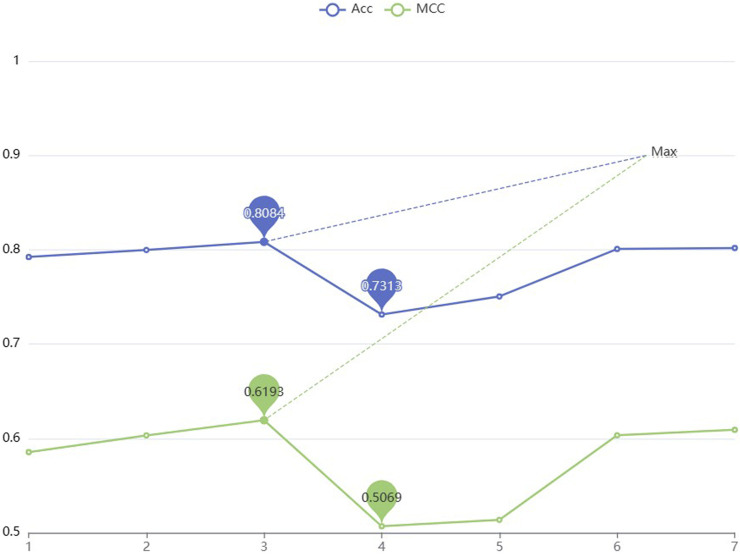
The comparison of Acc and MCC values for models with various amounts of denseblocks.

To improve the performance of the predictor, the parameters are optimized in two-branch residual connection DenseNet. In this section, different numbers of denseblocks are set and the Acc and MCC values are used to compare the model performance for different numbers of denseblocks. According to Figure 5, selecting a model with three denseblocks yields the highest performance. The trough is reached when the number is 4. After the number is 5, the Acc and MCC values increase as the amount of blocks grows, but considering that when the amount of denseblocks reaches 8, the maximum feature map scale in the model run has reached [211464,768], which is easy to cause insufficient server memory. Therefore, based on the consideration of computational complexity, the denseblock = 3 is selected.

### 3.2 Comparison of models with various encoding methods

**FIGURE 6:**
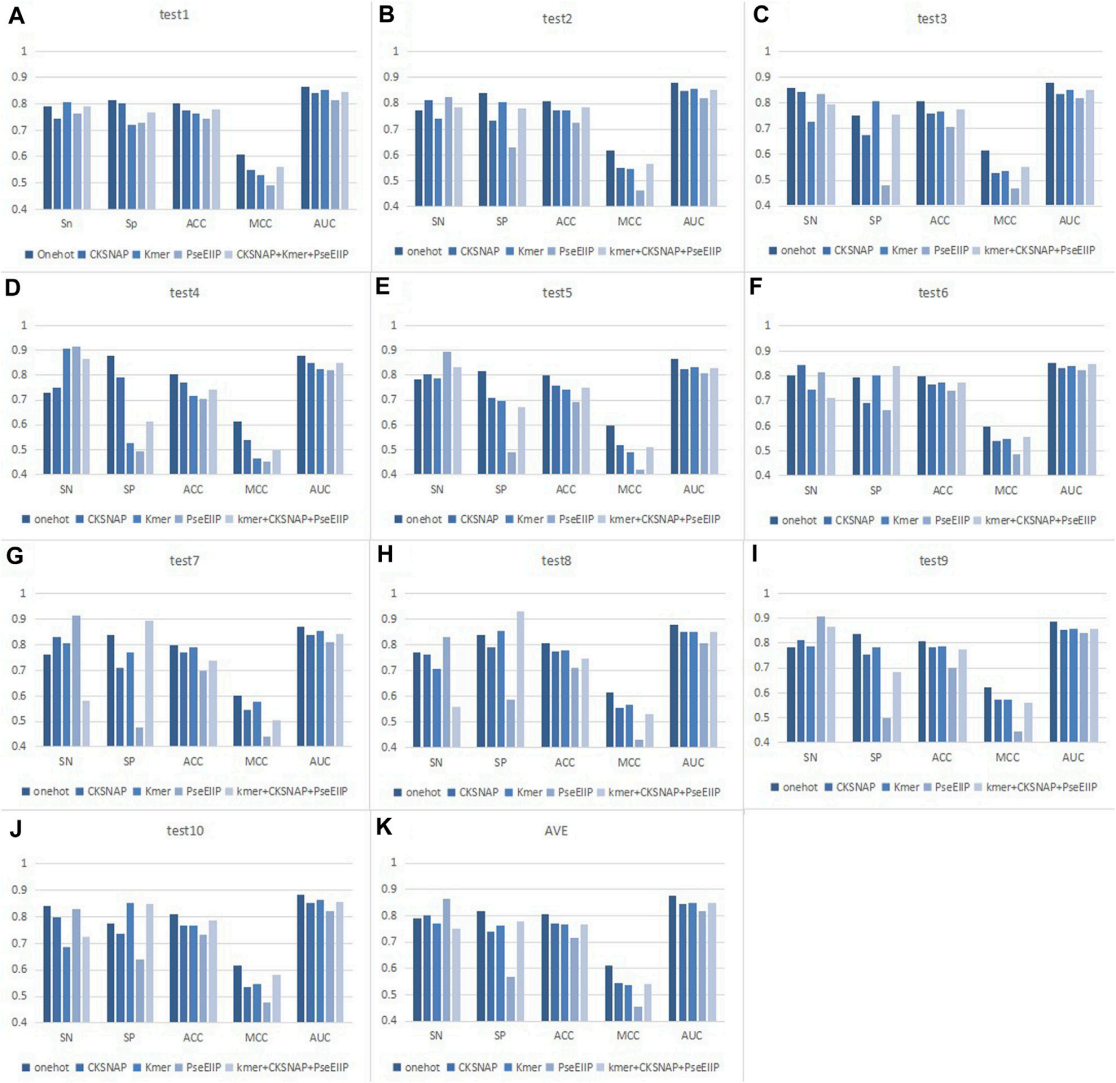
Performance comparison of different encoding methods on ten test sets. **(A–J)** Results for different test sets. **(K)** Average results of 10 test sets.

For the choice of feature coding methods, we considered four traditional coding methods, namely,: One-hot, composition of k-spaced nucleic acid pairs (CKSNAP), Kmer, and electron–ion interaction pseudopotentials of trinucleotide (PseEIIP). Also included: this coding scheme of CKSNAP + Kmer + PseEIIP combination. These coding methods have been applied in many studies (Li et al., 2020; Wang et al., 2021; Chen et al., 2021; El Allali et al., 2021; Le et al., 2022; Wang et al., 2023) and are not described in detail here. In addition to these traditional coding approaches, there are some new coding approaches including: Gene2vec (Zou et al., 2019), Geo2vec (Huang et al., 2022), Genomics features (Chen et al., 2019), Chemical property (Chen et al., 2017), Heuristic nucleotide physical-chemical properties reduction (Dou et al., 2020) also gave us a lot of inspiration on sequence encoding. We compared these coding schemes on ten balanced test datasets, as indicated in Figure 6. In the ten experiments, we tested univariate the encoding style suitable for the model using a unified classifier: two-branch residual connection DenseNet.

As seen in Figure 6, we can find that for each test set, the values of the indicators corresponding to the same coding method do not differ much. For example, the AUC value of the model with one-hot encoding in each dataset is about 0.87. The detailed performance of the different encoding methods of EMDL-ac4C on ten independent test sets is shown in Supplementary Tables S1–S10. This indicates that two-branch residual connection DenseNet has good generalizability and does not show excessive differences depending on the dataset. In addition, it is also obvious from Figure 6 that the model with one-hot coding has significantly higher Acc, MCC and AUC values than the other coding methods. After comprehensive consideration, we concluded that one-hot encoding is more suitable for two-branch residual connection DenseNet model for predicting ac4C sites.

### 3.3 Comparison of ensemble and non-ensemble models

**FIGURE 7:**
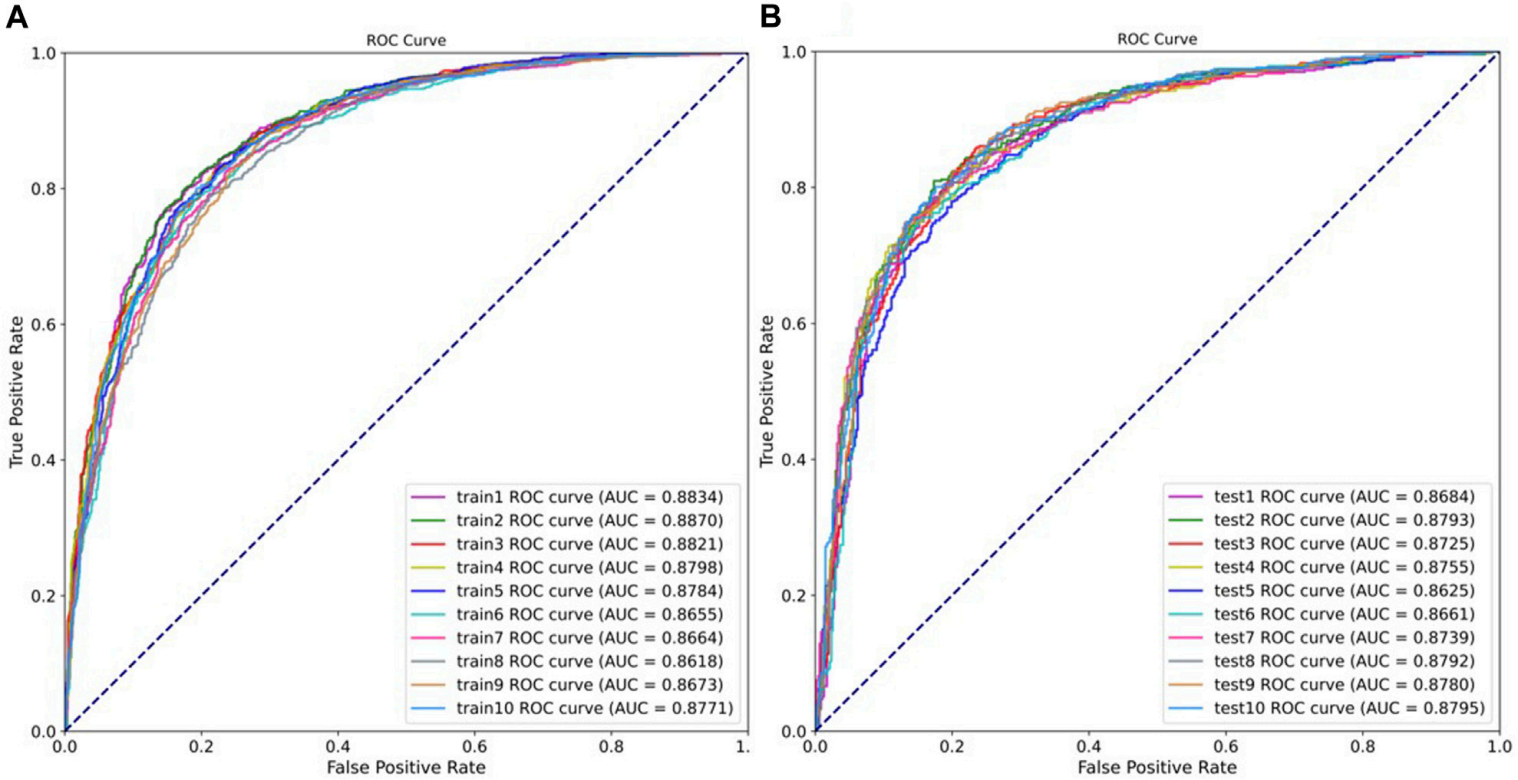
ROC curve of two-branch residual connection DenseNet on each dataset. **(A)** Results on training sets. **(B)** Results on testing sets.

**FIGURE 8:**
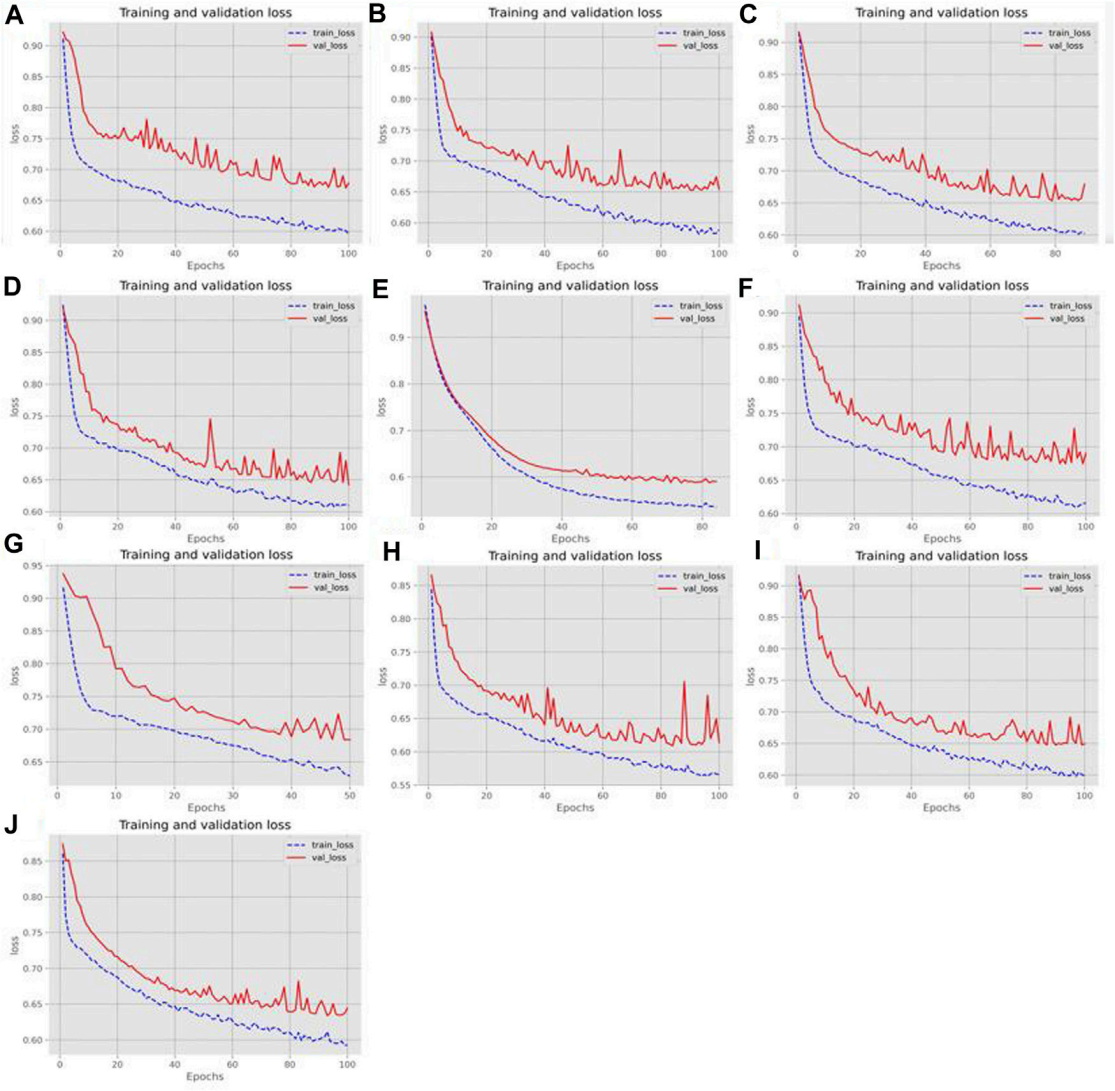
The loss change of the training and validation sets. **(A–J)** Loss changes in ten different training and validation sets.

To illustrate the effectiveness of the two-branch residual connection DenseNet more intuitively, we further tested its ROC curves on ten training and test sets, as shown in Figure 7. Also, Figure 8 showed the loss changes on the training and validation sets. The model demonstrated excellent performance and balanced results on ten training sets and ten testing sets. The difference in indicators on each dataset done not exceed 2.52%, indicating that the two branch residual connection DenseNet can stably predict the ac4C sites.

**FIGURE 9:**
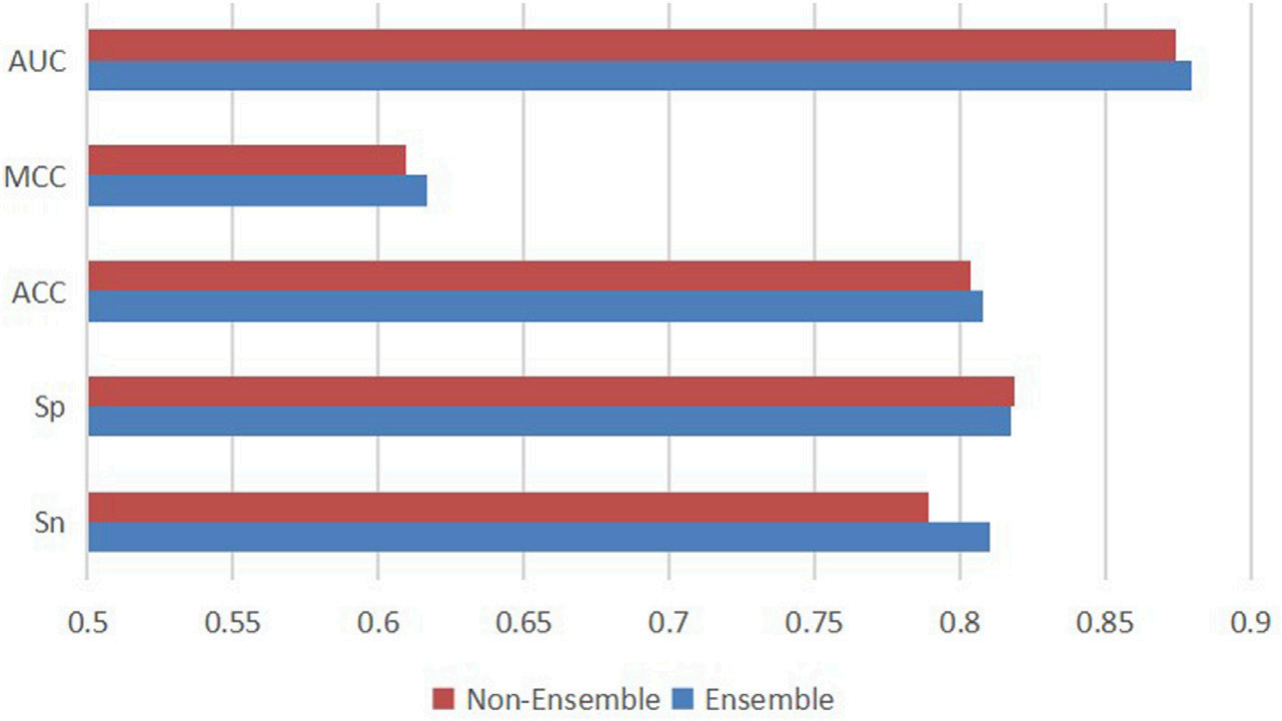
Performance comparison of single two-branch residual connection DenseNet and downsampling ensemble model EMDL-ac4C.

In addition, to research whether downsampling ensemble has better prediction ability, we made a comparison between the downsampling ensemble model and single two-branch residual connection DenseNet. On ten independent test sets, we used the ensemble classifier and the non-ensemble classifier to predict ac4C sites based on one-hot coding, and the average results were shown in Figure 9. The ensemble model EMDL-ac4C outperforms the non-ensemble model in Sn, Acc, MCC and AUC by 2.13%, 0.42%, 0.74% and 0.53%, respectively. In contrast, Sp is 0.14% lower, and collectively, the performance of the ensemble model is better than that of the non-ensemble model.

### 3.4 Comparison with other machine learning models

**FIGURE 10:**
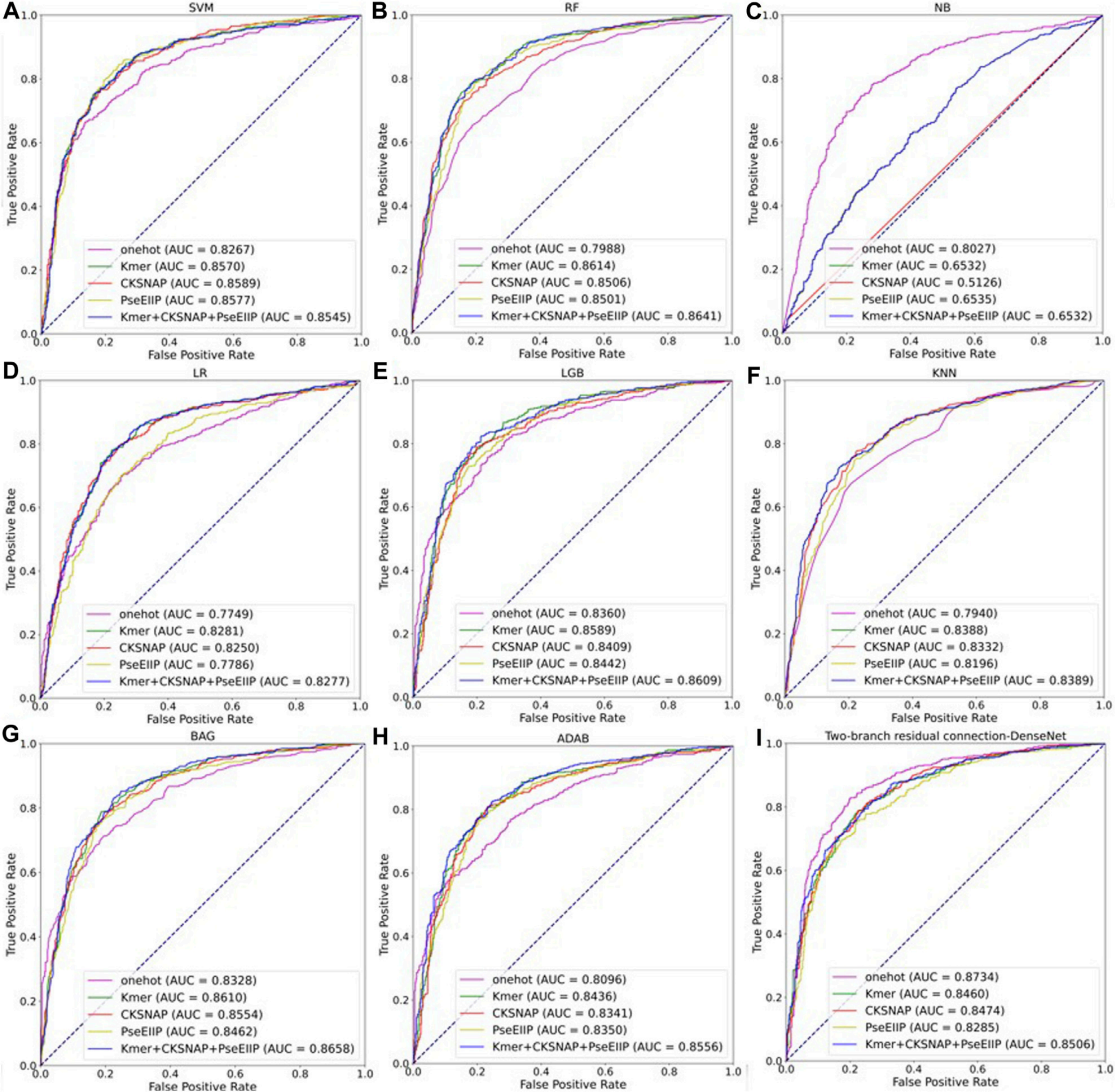
Multiple model ROC curves based on test sets with different encoding methods. **(A–H)** Results of machine learning. **(I)** Results of two-branch residual connection DenseNet.

To further evaluate the performance of the two-branch residual connection DenseNet model with that of other machine learning models, we performed a comparison of the average results of various coding methods and distinct classifiers on ten test datasets. The ROC curves comparison are shown in Figure 10, where, A-H are the machine learning models: support vector machine (SVM), random forest (RF), naive Bayesian (NB), logistic regression (LR), light gradient boosting machine (LGB), k-nearest neighbor (KNN), bagging (BAG) and adaboost classifier (ADAB). I is the result of two-branch residual connection DenseNet. It is clear from Figure 10 that among the machine learning models, SVM, LGB, and BAG perform well and NB performs the worst. Of course, the combination of the two-branch residual connection DenseNet and one-hot coding has the best performance. Table 2 shows the average performance metrics of EMDL-ac4C and additional machine learning models using one-hot coding on ten independent test sets. All four metrics of EMDL-ac4C return higher values than the other traditional machine learning models.

**TABLE 2:** Average performance comparison of EMDL-ac4C and additional machine learning models on ten independent test sets.

Model	Sn	Sp	MCC	AUC	Auprc
SVM	0.7610	0.7668	0.5279	0.8267	0.8301
RF	0.7248	0.7602	0.4854	0.7988	0.6824
NB	0.8046	0.6634	0.4956	0.8027	0.7904
LR	0.6998	0.6893	0.3892	0.7749	0.7626
LGB	0.7030	0.8081	0.5141	0.8360	0.8538
KNN	0.7786	0.6294	0.5028	0.7940	0.7873
DT	0.6998	0.6893	0.3892	0.625040	0.5783
BAG	0.7717	0.740814	0.5427	0.8328	0.8557
ADAB	0.6833	0.7636	0.4486	0.8096	0.8453
EMDL-ac4C	**0.8104**	**0.8173**	**0.6169**	**0.8734**	**0.8643**

The best outcomes are in bold.

**FIGURE 11:**
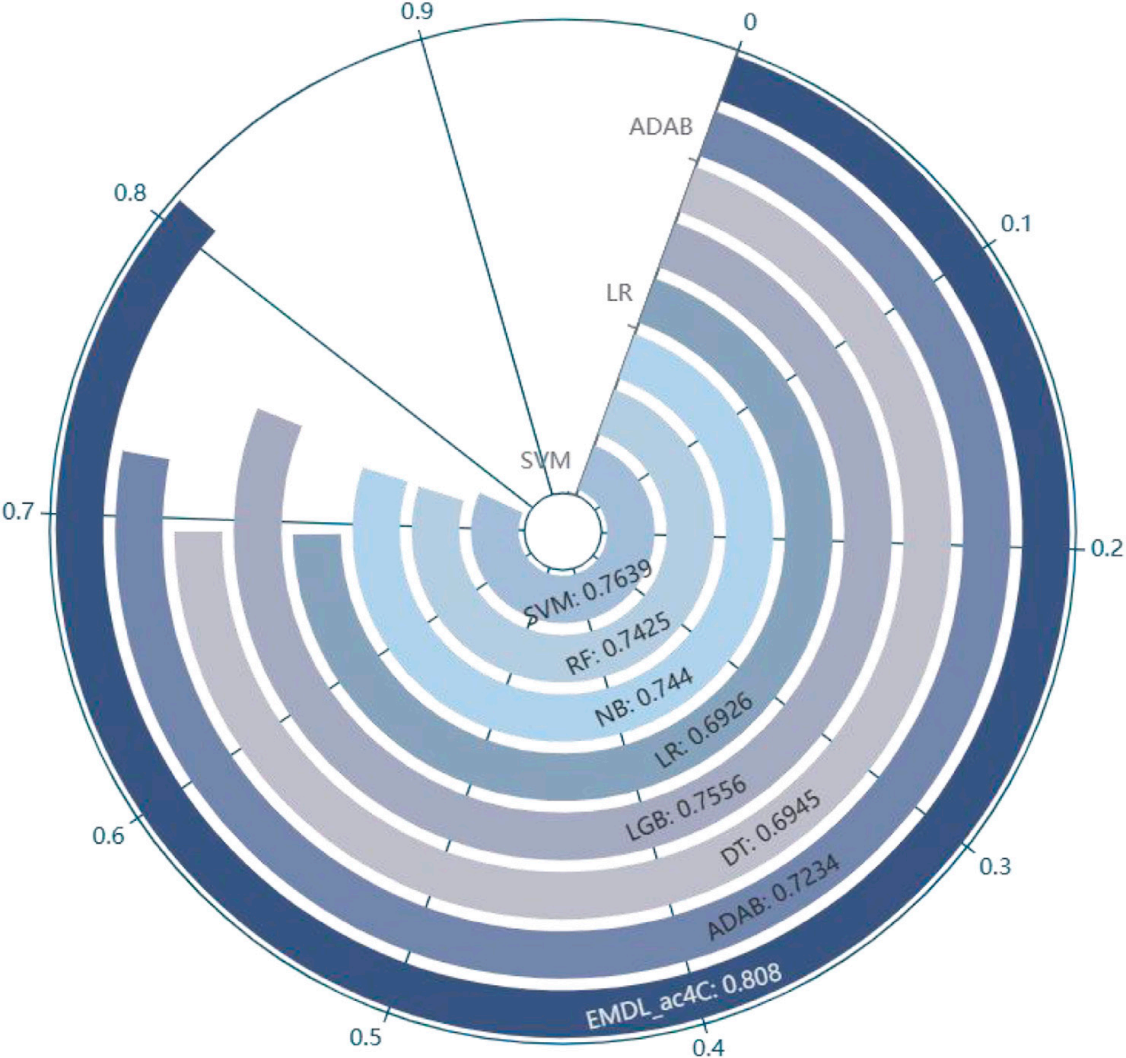
Comparison of Average Accuracy of EMDL-ac4C and additional machine learning models on ten independent test sets.

In balanced datasets, accuracy (Acc) is one of the important metrics to evaluate the classifier performance. Figure 11 visualizes that EMDL-ac4C is more effective than other machine learning models.

### 3.5 Comparison with other advanced models

To assess the prediction performance of EMDL-ac4C, we compared EMDL-ac4C with several advanced models for analysis, including: Cross Stage Partial DenseNet (CSPNet) (Wang et al., 2020), VGG-16 (Guan et al., 2019), ResNet (He et al., 2016), VGG-19 (Xiao et al., 2020), Inception V3 (Yu et al., 2017). These models performed differently on the ten balanced test sets, as shown in Supplementary Tables S12–S17, while the average prediction results on the ten datasets are shown in Table 3. VGG-16 scored the highest in Sn, but the score of Sp was too low, and the balance between Sn and Sp was lost, with too much deviation, and the prediction accuracy was not high. In contrast, our model EMDL-ac4C reached a balance between Sn and Sp with less than 1% deviation, and obtained the highest Sp, MCC, Acc, and AUC scores among several models, getting the greatest prediction results.

**TABLE 3:** Average performance of several advanced models on ten test sets.

Model	Sn	Sp	MCC	Acc	AUC
VGG-16	**0.9227**	0.2921	0.2533	0.6074	0.6850
ResNet	0.6673	0.6285	0.3008	0.6479	0.7086
CSPNet	0.8267	0.7349	0.5683	0.7808	0.8576
VGG-19	0.7537	0.6417	0.4304	0.6977	0.8231
Inception V3	0.7934	0.3488	0.1770	0.5711	0.6596
EMDL-ac4C	0.8104	**0.8173**	**0.6169**	**0.8080**	**0.8794**

The best outcomes are in bold.

To further evaluate the predictive performance of EMDL-ac4C, we collected three ac4C sites identified by Oxford Nanopore Technology (ONT) from the large public database DirectRMDB (Zhang. et al., 2022) as an additional test set, these three ac4C loci are located at positions 453630, 455959, and 456452 of chromosome NC_001144.5, respectively. After testing, all three ac4C loci were correctly predicted, and the probabilities of predicting positive samples were 0.7923848, 0.9826068, and 0.9666126, respectively. Therefore, we can consider EMDL-ac4C as a high-performance ac4C classifier.

### 3.6 Comparison of different classifiers

**FIGURE 12:**
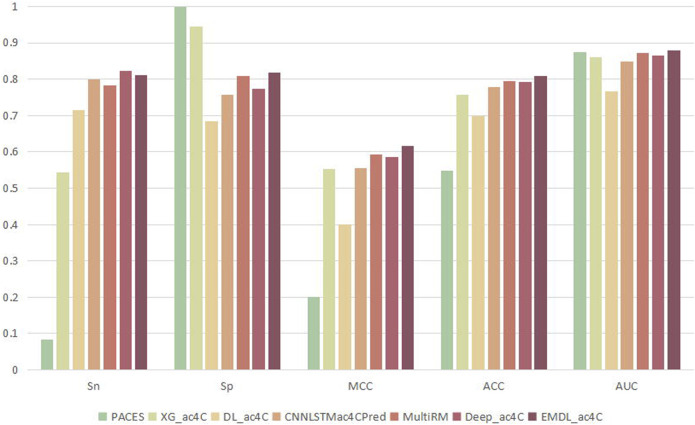
Comparison of the average results of ten test sets on various predictors.

To prove the validity of EMDL-ac4C, we found six models that can be used to predict ac4C loci for comparison, including PACES (Zhao et al., 2019a), XG_ac4C (Alam et al., 2020b), DL_ac4C (Iqbal et al., 2022), CNNLSTMac4CPred(Zhang et al., 2022), MultiRM (Song et al., 2021) and DeepAc4C (Wang et al., 2021). Among these six predictors, PACES and XG_ac4C used machine learning approaches: random forest, XGboost. While DL_ac4C, CNNLSTMac4CPred, MultiRMand DeepAc4C used the deep learning approach. For a fair comparison, all seven predictors were tested using the same training and testing sets, and the predicted results were compared to determine their performance. Figure 12 had shown the average result of the seven predictors on ten test sets. Among them, EMDL-ac4C had the best comprehensive performance, followed by the deep learning model MultiRM, and then DeepAc4C, CNNLSTMac4CPred, XG_ac4C and DL_ac4C. PACES had the worst performance. Compared to MultiRM, EMDL-ac4C was 2.88%, 0.79%, 2.33%, 1.25% and 0.85% higher for Sn, Sp, MCC, ACC, and AUC, respectively. Meanwhile, compared to DeepAc4C, EMDL-ac4C was 4.39%, 3.12%, 1.61%, and 1.45% higher in Sp, MCC, Acc, and AUC, respectively. In addition, EMDL-ac4C was also higher than CNNLSTMac4CPred in all metrics, and the average is 3.93% higher. For Sp, PACES and XG_ac4C had higher return values than EMDL-ac4C. Nevertheless, in particular, the difference between the Sn and Sp values of PACES and XG_ac4C was too large, even reaching 91.52% for PACES, and too low Sn indicated that few positive samples were identified, which was not a good phenomenon. For the balanced dataset, we pay more attention to the return value of Acc, and the larger the Acc, the better the model performance. Our model EMDL-ac4C had higher Acc metrics than DL_ac4C, XG_ac4C and PACES: 10.89%, 5.08% and 26.08% higher, respectively. DeepAc4C is the most advanced model at present, so the detailed analysis of EMDL-ac4C and DeepAc4C is compared, see Table 4.

**TABLE 4:** The performance of DeepAc4C and EMDL-ac4C.

Dataset	Model	Training Acc	Validation Acc	Test Acc	Test MCC	Test AUC
D1	DeepAc4C	0.8093	0.8043	0.7934	0.5871	0.8620
	EMDL-ac4C	**0.8911**	**0.8092**	**0.8104**	**0.6217**	**0.8821**
D2	DeepAc4C	0.8195	0.8000	0.7943	0.5921	0.8660
	EMDL-ac4C	**0.8967**	**0.8075**	**0.8126**	**0.6267**	**0.8801**
D3	DeepAc4C	0.8209	0.8043	0.7874	0.5777	0.8641
	EMDL-ac4C	**0.8985**	**0.8097**	**0.8115**	**0.6244**	**0.8832**
D4	DeepAc4C	0.8490	**0.8348**	0.7969	0.5945	0.8658
	EMDL-ac4C	**0.8954**	0.8096	**0.8040**	**0.6102**	**0.8819**
D5	DeepAc4C	0.8403	0.8043	0.7950	0.5902	0.8646
	EMDL-ac4C	**0.8919**	**0.8063**	**0.8019**	**0.6041**	**0.8647**
D6	DeepAc4C	0.7996	0.7913	0.7938	0.5897	0.8645
	EMDL-ac4C	**0.8871**	**0.8076**	**0.7997**	**0.6000**	**0.8665**
D7	DeepAc4C	0.8209	**0.8609**	0.7911	0.5836	0.8671
	EMDL-ac4C	**0.8915**	0.8179	**0.7976**	**0.5956**	**0.8749**
D8	DeepAc4C	0.8475	0.8043	0.7978	0.5959	0.8657
	EMDL-ac4C	**0.8880**	**0.8056**	**0.8147**	**0.6306**	**0.8809**
D9	DeepAc4C	0.8078	0.8130	0.7846	0.5703	0.8615
	EMDL-ac4C	**0.8308**	**0.8177**	**0.8169**	**0.6340**	**0.8908**
D10	DeepAc4C	0.8277	**0.8217**	0.7850	0.5725	0.8680
	EMDL-ac4C	**0.8924**	0.8201	**0.8147**	**0.6295**	**0.8913**
Average	DeepAc4C	0.8242	**0.8139**	0.7919	0.5857	0.8649
	EMDL-ac4C	**0.8927**	0.8111	**0.8084**	**0.6177**	**0.8794**

The best outcomes are in bold.

Some of the classification performance metrics of EMDL-ac4C and DeepAc4C on the training set, validation set, and test set were listed in Table 4 with comparative analysis of the two predictors. EMDL-ac4C had a greater accuracy (Acc) than DeepAc4C in all 10 balanced datasets used for training. Among them, the best performance was on D6, which was 8.75% higher. On the validation dataset, EMDL-ac4C performed relatively poorly on the D4, D7 datasets, and it also performed slightly worse than DeepAc4C on D10. In this regard, we believe that EMDL-ac4C is unable to learn enough information and features from the data due to the small number of data in the validation set, which affects the performance of the model. For the datasets in this paper, the samples used for training in each balanced dataset were 2066, while the amounts of samples used for validation and testing were 230 and 934, correspondingly.

For the ten balanced test sets, EMDL-ac4C obtained good prediction results, with the Acc, MCC and AUC values of each test subset exceeding the corresponding metrics of DeepAc4C. The average Acc of the ten test sets of EMDL-ac4C was 1.61% higher than the average Acc of DeepAc4C, and the average MCC and average AUC values were 3.12% and 1.45% higher, respectively. This showed that the EMDL-ac4C model is effective and also has good generalization. In addition, it can also be seen from Table 4 that the difference of each metric corresponding to the ten balanced data sets is small, which proven that EMDL-ac4C is a stable and reliable model. By comparison, see Supplementary Tables S14, S15, the MCC values of VGG16 and VGG 19 models on different equilibrium datasets vary widely. VGG 16 had a MCC value of 0.99% on D7, and the MCC value on D1 reaches 52.75%, with a variance of 51.76%. The maximum difference in MCC values for VGG19 was also as high as 42.63%, which indicated that the VGG model is not stable on the data sets of this paper.

To evaluate the performance of our model on the unbalanced dataset, we use the dataset downloaded from the PACES website (http://www.rnanut.net/paces/) for testing. In this case, the training set contains 1,160 positive and 10,855 negative samples, respectively, while the test set contains 469 positive and 4,343 negative samples. The results of the 5-fold cross-validation and independent tests of our base model Two-branch residual connection DenseNet and two other predictors PACES (Zhao et al., 2019b) and XG-ac4C (Alam et al., 2020a) are shown in Table 5.

**TABLE 5:** The AUC values and Aupr values of PACES, XG-ac4C and Two-branch residual connection DenseNet.

Dataset	Methods	AUC	Aupr
Cross-validation	PACES	0.885	0.559
	XG-ac4C	**0.910**	**0.653**
	Two-branch residual connection DenseNet	0.904	0.615
Independent-test	PACES	0.874	0.485
	XG-ac4C	0.889	0.581
	Two-branch residual connection DenseNet	**0.901**	**0.594**

The best outcomes are in bold.

On cross-validation, the AUC value of Two-branch residual connection DenseNet is slightly lower than XG-ac4C, but exceeds PACES, and the same is true for the Aupr value, which may be due to the fact that Two-branch residual connection DenseNet is only the base model, without unbalanced preprocessing of the dataset and without using the ensemble method. In the independent test, Two-branch residual connection DenseNet is better than the other two models, and the difference between the independent test and cross-validation results is small, not more than 0.021, and there is no overfitting, which just shows that Two-branch residual connection DenseNet has a strong generalization ability.

### 3.7 Visualization of the classification ability of EMDL-ac4C

**FIGURE 13:**
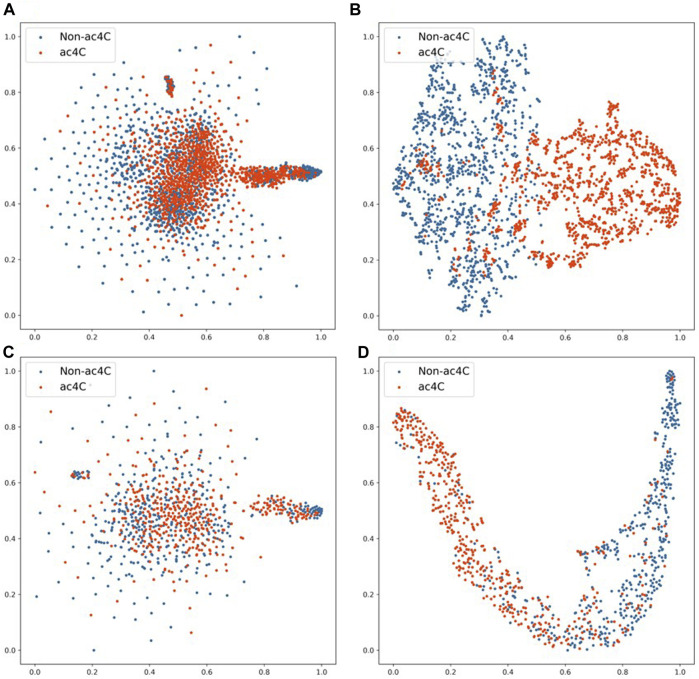
2D t-SNE visualization of the training and testing sets. **(A)** 2D t-SNE visualization of the training set with one-hot encoding. **(B)** 2D t-SNE visualization of features learned from the training set by EMDL-ac4C. **(C)** 2D t-SNE visualization of the testing set with one-hot encoding. **(D)** 2D t-SNE visualization of features learned from the testing set by EMDL-ac4C.

To test the classification performance of EMDL-ac4C, we selected D1 in ten balanced datasets for validation. After encoding the sequence data with one-hot, We reduced the encoding vector to two-dimensional using t-distribution random neighbor embedding (t-SNE) (van der Maaten and Hinton, 2008) method, as shown in Figures 13A, C, where (A) and (C) were the classification effects of the training and testing sets after one-hot encoding using t-SNE downscaling, respectively. In order to compare with (A) (C) in Figure 13, we first extracted the important features from the training and testing data after one-hot encoding using EMDL-ac4C, and then downscaled them by t-SNE, and finally displayed the classification effects as shown in Figures 13B, D. The red circles in Figure 13 stand for the positive class samples, whereas the blue circles for the negative class samples. From Figure 13, we can clearly see that there are more overlapping clusters generated after one-hot coding, which indicates that the quality of one-hot coding is imperfect. In contrast, after EMDL-ac4C extracts the features, fewer overlapping clusters are generated, especially the two classes of clusters generated in the testing set have reached a highly disjoint classification effect, which proves the efficiency of EMDL-ac4C in terms of extracting features, that is, EMDL-ac4C has powerful classification ability.

## 4 Conclusion

In this work, we built a downsampling ensemble learning model called EMDL-ac4C, which aimed to predict ac4C sites from sequence fragments of RNA. To effectively identify the ac4C locus, we had done a lot of work at both sequence encoding and feature extraction levels. Firstly, we had compared five commonly used feature encoding schemes and found that the combination of simple one-hot encoding and deep learning models can identify ac4C loci more efficiently. Second, we proposed the ensemble learning model EMDL-ac4C to extract features and predict sites, whose underlying learner was two-branch residual connection DenseNet. Compared with other advanced models and predictors for identifying ac4C, EMDL-ac4C obtained superior performance in independent tests, which proved EMDL-ac4C’s powerful feature learning capability and predictive power. We will develop the model and increase its prediction power in subsequent studies. For instance, we will be able to anticipate multi-class sites simultaneously, such as 6ma, 4mc, *etc.*

## Data Availability

The original contributions presented in the study are included in the article/Supplementary Material, further inquiries can be directed to the corresponding authors.
