# Supplementary material for: EMDL-ac4C: identifying N4-acetylcytidine based on ensemble two-branch residual connection DenseNet and attention
Source: Front Genet. 2023 Jul 13;14:1232038. doi: 10.3389/fgene.2023.1232038 (PMC10372626; doi:10.3389/fgene.2023.1232038)
Supplement: Supplementary file 1 [file Table1.DOCX]

**Supplementary Material**

# EMDL-ac4C: identifying n4-acetylcytidine based on ensemble two-branch residual connection DenseNet and attention

Jianhua Jia^1*^, Zhangying Wei ^1*^, Xiaojing Cao ^1^

^1^School of Information Engineering, Jingdezhen Ceramic University, Jingdezhen 333403, China

*** Correspondence:**

Jianhua Jia: [jjh163yx@163.com](mailto:jjh163yx@163.com)

Zhangying Wei: [weizy5003@163.com](mailto:weizy5003@163.com)

## Performance of different encoding methods of EMDL_ac4C on ten independent test sets

**Table 1.** Performance of different encoding methods of EMDL_ac4C on test1.

| **Test1** | Sn | Sp | ACC | MCC | AUC |
| --- | --- | --- | --- | --- | --- |
| onehot | 0.7923 | **0.8137** | **0.8029** | **0.6061** | **0.8652** |
| CKSNAP | 0.7452 | 0.8030 | 0.7741 | 0.5491 | 0.8425 |
| Kmer | **0.8073** | 0.7195 | 0.7634 | 0.5288 | 0.8540 |
| PseEIIP | 0.7623 | 0.7281 | 0.7452 | 0.4907 | 0.8138 |
| kmer+CKSNAP+PseEIIP | 0.7902 | 0.7687 | 0.7794 | 0.5591 | 0.8468 |

**Table 2.** Performance of different encoding methods of EMDL_ac4C on test2.

| **Test2** | Sn | Sp | ACC | MCC | AUC |
| --- | --- | --- | --- | --- | --- |
| onehot | 0.7730 | **0.8415** | **0.8072** | **0.6160** | **0.8816** |
| CKSNAP | 0.8137 | 0.7323 | 0.7730 | 0.5479 | 0.8484 |
| Kmer | 0.7409 | 0.8051 | 0.7730 | 0.5472 | 0.8561 |
| PseEIIP | **0.8223** | 0.6296 | 0.7259 | 0.4605 | 0.8189 |
| kmer+CKSNAP+PseEIIP | 0.7837 | 0.7816 | 0.7827 | 0.5653 | 0.8510 |

**Table 3.** Performance of different encoding methods of EMDL_ac4C on test3.

| **Test3** | Sn | Sp | ACC | MCC | AUC |
| --- | --- | --- | --- | --- | --- |
| onehot | 0.8608 | 0.7516 | **0.8062** | **0.6161** | **0.8793** |
| CKSNAP | 0.8437 | 0.6767 | 0.7602 | 0.5278 | 0.8367 |
| Kmer | 0.7281 | **0.8073** | 0.7677 | 0.5370 | 0.8528 |
| PseEIIP | **0.9358** | 0.4796 | 0.7077 | 0.4668 | 0.8199 |
| kmer+CKSNAP+PseEIIP | 0.7944 | 0.7559 | 0.7752 | 0.5507 | 0.8498 |

**Table 4.** Performance of different encoding methods of EMDL_ac4C on test4.

| **Test4** | Sn | Sp | ACC | MCC | AUC |
| --- | --- | --- | --- | --- | --- |
| onehot | 0.7281 | **0.8779** | **0.8029** | **0.6129** | **0.8784** |
| CKSNAP | 0.7495 | 0.7902 | 0.7698 | 0.5401 | 0.8499 |
| Kmer | 0.9058 | 0.5246 | 0.7152 | 0.4656 | 0.8243 |
| PseEIIP | **0.9143** | 0.4946 | 0.7045 | 0.4506 | 0.8219 |
| kmer+CKSNAP+PseEIIP | 0.8672 | 0.6124 | 0.7398 | 0.4960 | 0.8499 |

**Table 5.** Performance of different encoding methods of EMDL_ac4C on test5.

| **Test5** | Sn | Sp | ACC | MCC | AUC |
| --- | --- | --- | --- | --- | --- |
| onehot | 0.7816 | **0.8158** | **0.7987** | **0.5977** | **0.8660** |
| CKSNAP | 0.8051 | 0.7088 | 0.7570 | 0.5163 | 0.8256 |
| Kmer | 0.7880 | 0.6981 | 0.7430 | 0.4881 | 0.8319 |
| PseEIIP | **0.8951** | 0.4882 | 0.6916 | 0.4196 | 0.8087 |
| kmer+CKSNAP+PseEIIP | 0.8308 | 0.6724 | 0.7516 | 0.5097 | 0.8277 |

**Table 6.** Performance of different encoding methods of EMDL_ac4C on test6.

| **Test6** | Sn | Sp | ACC | MCC | AUC |
| --- | --- | --- | --- | --- | --- |
| onehot | 0.8009 | 0.7944 | **0.7976** | **0.5953** | **0.8515** |
| CKSNAP | **0.8415** | 0.6916 | 0.7666 | 0.5393 | 0.8326 |
| Kmer | 0.7452 | 0.8009 | 0.7730 | 0.5469 | 0.8395 |
| PseEIIP | 0.8158 | 0.6638 | 0.7398 | 0.4853 | 0.8243 |
| kmer+CKSNAP+PseEIIP | 0.7109 | **0.8394** | 0.7752 | 0.5549 | 0.8461 |

**Table 7.** Performance of different encoding methods of EMDL_ac4C on test7.

| **Test7** | Sn | Sp | ACC | MCC | AUC |
| --- | --- | --- | --- | --- | --- |
| onehot | 0.7602 | 0.8373 | **0.7987** | **0.5992** | **0.8710** |
| CKSNAP | 0.8287 | 0.7109 | 0.7698 | 0.5434 | 0.8369 |
| Kmer | 0.8073 | 0.7709 | 0.7891 | 0.5785 | 0.8531 |
| PseEIIP | **0.9165** | 0.4775 | 0.6970 | 0.4385 | 0.8105 |
| kmer+CKSNAP+PseEIIP | 0.5824 | **0.8951** | 0.7388 | 0.5027 | 0.8445 |

**Table 8.** Performance of different encoding methods of EMDL_ac4C on test8.

| **Test8** | Sn | Sp | ACC | MCC | AUC |
| --- | --- | --- | --- | --- | --- |
| onehot | 0.7709 | 0.8415 | **0.8062** | **0.6139** | **0.8796** |
| CKSNAP | 0.7623 | 0.7923 | 0.7773 | 0.5549 | 0.8508 |
| Kmer | 0.7088 | 0.8544 | 0.7816 | 0.5692 | 0.8529 |
| PseEIIP | **0.8330** | 0.5867 | 0.7099 | 0.4330 | 0.8066 |
| kmer+CKSNAP+PseEIIP | 0.5610 | **0.9315** | 0.7463 | 0.5302 | 0.8501 |

**Table 9.** Performance of different encoding methods of EMDL_ac4C on test9.

| **Test9** | Sn | Sp | ACC | MCC | AUC |
| --- | --- | --- | --- | --- | --- |
| onehot | 0.7816 | **0.8373** | **0.8094** | **0.6198** | **0.8848** |
| CKSNAP | 0.8137 | 0.7559 | 0.7848 | 0.5705 | 0.8546 |
| Kmer | 0.7880 | 0.7837 | 0.7859 | 0.5717 | 0.8587 |
| PseEIIP | **0.9058** | 0.4989 | 0.7024 | 0.4430 | 0.8402 |
| kmer+CKSNAP+PseEIIP | 0.8651 | 0.6852 | 0.7752 | 0.5594 | 0.8584 |

**Table 10.** Performance of different encoding methods of EMDL_ac4C on test10.

| **Test10** | Sn | Sp | ACC | MCC | AUC |
| --- | --- | --- | --- | --- | --- |
| onehot | 0.8415 | 0.7752 | **0.8083** | **0.6180** | **0.8832** |
| CKSNAP | 0.7966 | 0.7366 | 0.7666 | 0.5342 | 0.8518 |
| Kmer | 0.6852 | **0.8522** | 0.7687 | 0.5451 | 0.8652 |
| PseEIIP | **0.8287** | 0.6381 | 0.7334 | 0.4755 | 0.8227 |
| kmer+CKSNAP+PseEIIP | 0.7259 | 0.8501 | 0.7880 | 0.5805 | 0.8558 |

**Table 11.** Average performance of different encoding methods of EMDL_ac4C on ten test sets.

| **AVE** | Sn | Sp | ACC | MCC | AUC |
| --- | --- | --- | --- | --- | --- |
| onehot | 0.7891 | **0.8187** | **0.8038** | **0.6095** | **0.8741** |
| CKSNAP | 0.8001 | 0.7398 | 0.7699 | 0.5424 | 0.8430 |
| Kmer | 0.7705 | 0.7617 | 0.7661 | 0.5378 | 0.8489 |
| PseEIIP | **0.86296** | 0.56851 | 0.71574 | 0.45635 | 0.81875 |
| kmer+CKSNAP+PseEIIP | 0.75116 | 0.77923 | 0.76522 | 0.54085 | 0.84801 |

## Performance of several advanced models on different test sets

**Table 12.** ResNet's performance on ten test sets.

| **ResNet** | Sn | Sp | ACC | MCC | AUC |
| --- | --- | --- | --- | --- | --- |
| test 1 | 0.5268 | 0.7966 | 0.6617 | 0.3358 | 0.7067 |
| test 2 | 0.6702 | 0.5696 | 0.6199 | 0.2411 | 0.6861 |
| test 3 | 0.6767 | 0.606 | 0.6413 | 0.2834 | 0.6893 |
| test 4 | 0.6831 | 0.5974 | 0.6403 | 0.2815 | 0.7071 |
| test 5 | 0.8266 | 0.409 | 0.6178 | 0.2592 | 0.7011 |
| test 6 | 0.6488 | 0.6188 | 0.6338 | 0.2678 | 0.6996 |
| test 7 | 0.7559 | 0.5546 | 0.6552 | 0.317 | 0.7129 |
| test 8 | 0.6403 | 0.696 | 0.6681 | 0.3367 | 0.7235 |
| test 9 | 0.606 | 0.7495 | 0.6777 | 0.3592 | 0.742 |
| test 10 | 0.6381 | 0.6874 | 0.6627 | 0.3259 | 0.7173 |
| Ave | 0.6673 | 0.6285 | 0.6479 | 0.3008 | 0.7086 |

**Table 13.** Inception V3's performance on ten test sets.

| **Inception V3** | Sn | Sp | ACC | MCC | AUC |
| --- | --- | --- | --- | --- | --- |
| test 1 | 0.7516 | 0.5931 | 0.6724 | 0.3492 | 0.7527 |
| test 2 | 0.818 | 0.5332 | 0.6756 | 0.3663 | 0.761 |
| test 3 | 0.5846 | 0.8116 | 0.6981 | 0.4068 | 0.7751 |
| test 4 | 0.3469 | 0.9079 | 0.6274 | 0.3078 | 0.7249 |
| test 5 | 0.8051 | 0.5696 | 0.6874 | 0.3856 | 0.7514 |
| test 6 | 0.8137 | 0.5375 | 0.6756 | 0.3654 | 0.7501 |
| test 7 | 0.5739 | 0.773 | 0.6734 | 0.354 | 0.7417 |
| test 8 | 0.5482 | 0.8179 | 0.6831 | 0.3803 | 0.7564 |
| test 9 | 0.6317 | 0.7302 | 0.6809 | 0.3637 | 0.7429 |
| test 10 | 0.6017 | 0.7752 | 0.6884 | 0.3827 | 0.7533 |
| Ave | 0.6475 | 0.7049 | 0.6762 | 0.3662 | 0.7510 |

**Table 14.** VGG16's performance on ten test sets.

| **VGG16** | Sn | Sp | ACC | MCC | AUC |
| --- | --- | --- | --- | --- | --- |
| test 1 | 0.6017 | 0.9015 | 0.7516 | 0.5275 | 0.8457 |
| test 2 | 0.9807 | 0.0642 | 0.5225 | 0.1124 | 0.6073 |
| test 3 | 0.9979 | 0.0086 | 0.5032 | 0.044 | 0.5159 |
| test 4 | 0.9786 | 0.0921 | 0.5353 | 0.1527 | 0.6864 |
| test 5 | 0.9036 | 0.5696 | 0.7366 | 0.5021 | 0.8284 |
| test 6 | 0.9251 | 0.5289 | 0.7269 | 0.4944 | 0.8322 |
| test 7 | 0.9893 | 0.0128 | 0.5011 | 0.0099 | 0.5825 |
| test 8 | 0.9807 | 0.0257 | 0.5032 | 0.0217 | 0.5316 |
| test 9 | 0.9850 | 0.0535 | 0.5193 | 0.1059 | 0.5606 |
| test 10 | 0.8840 | 0.6638 | 0.7741 | 0.562 | 0.8590 |
| Ave | 0.9227 | 0.2921 | 0.6074 | 0.2533 | 0.6850 |

**Table 15.** VGG19's performance on ten test sets.

| **VGG19** | Sn | Sp | ACC | MCC | AUC |
| --- | --- | --- | --- | --- | --- |
| test 1 | 0.6874 | 0.8329 | 0.7601 | 0.5259 | 0.8339 |
| test 2 | 0.9379 | 0.349 | 0.6434 | 0.355 | 0.8115 |
| test 3 | 0.3854 | 0.9443 | 0.6648 | 0.3976 | 0.8275 |
| test 4 | 0.7366 | 0.8029 | 0.7698 | 0.5408 | 0.8447 |
| test 5 | 0.9657 | 0.1905 | 0.5781 | 0.2474 | 0.7509 |
| test 6 | 0.8458 | 0.6531 | 0.7494 | 0.5084 | 0.8304 |
| test 7 | 0.6531 | 0.8436 | 0.7483 | 0.506 | 0.8264 |
| test 8 | 0.6616 | 0.8822 | 0.7719 | 0.5576 | 0.8472 |
| test 9 | 0.9807 | 0.0749 | 0.5278 | 0.1313 | 0.8135 |
| test 10 | 0.6831 | 0.8436 | 0.7633 | 0.5336 | 0.8445 |
| Ave | 0.7537 | 0.6417 | 0.6977 | 0.4304 | 0.8231 |

**Table 16.** CSPNet's performance on ten test sets.

| **CSPNet** | Sn | Sp | ACC | MCC | AUC |
| --- | --- | --- | --- | --- | --- |
| test 1 | 0.7644 | 0.8286 | 0.7965 | 0.5943 | 0.8549 |
| test 2 | 0.7858 | 0.8137 | 0.7997 | 0.5998 | 0.8631 |
| test 3 | 0.9057 | 0.6124 | 0.7591 | 0.5421 | 0.8572 |
| test 4 | 0.7516 | 0.8372 | 0.7944 | 0.591 | 0.8611 |
| test 5 | 0.8201 | 0.7302 | 0.7752 | 0.5525 | 0.8342 |
| test 6 | 0.8651 | 0.6338 | 0.7494 | 0.5128 | 0.8494 |
| test 7 | 0.8501 | 0.6895 | 0.7698 | 0.5467 | 0.8509 |
| test 8 | 0.8693 | 0.6745 | 0.7719 | 0.5545 | 0.8663 |
| test 9 | 0.8651 | 0.7302 | 0.7976 | 0.6007 | 0.8684 |
| test 10 | 0.7901 | 0.7987 | 0.7944 | 0.5888 | 0.8708 |
| Ave | 0.8267 | 0.7349 | 0.7808 | 0.5683 | 0.8576 |

**Table 17.** EMDL_ac4C's performance on ten test sets.

| **EMDL_ac4C** | Sn | Sp | ACC | MCC | AUC |
| --- | --- | --- | --- | --- | --- |
| test 1 | 0.7859 | 0.8351 | 0.8104 | 0.6217 | 0.8800 |
| test 2 | 0.7623 | 0.8544 | 0.8083 | 0.6193 | 0.8801 |
| test 3 | 0.8437 | 0.7794 | 0.8115 | 0.6244 | 0.8832 |
| test 4 | 0.8051 | 0.8201 | 0.8126 | 0.6253 | 0.8818 |
| test 5 | 0.7859 | 0.8180 | 0.8019 | 0.6041 | 0.8647 |
| test 6 | 0.7794 | 0.8201 | 0.7997 | 0.6001 | 0.8665 |
| test 7 | 0.8158 | 0.7794 | 0.7976 | 0.5956 | 0.8749 |
| test 8 | 0.7859 | 0.8437 | 0.8147 | 0.6306 | 0.8809 |
| test 9 | 0.8308 | 0.8030 | 0.8169 | 0.6340 | 0.8908 |
| test 10 | 0.8094 | 0.8201 | 0.8147 | 0.6295 | 0.8913 |
| Ave | 0.8104 | 0.8173 | 0.8080 | 0.6169 | 0.8794 |
